# A Promise Without Panacea: Psychedelic-Assisted Therapies in Modern Psychiatry

**DOI:** 10.7759/cureus.102421

**Published:** 2026-01-27

**Authors:** Mohsin Raza, Jasleen Kaur

**Affiliations:** 1 Psychiatry, Dover Behavioral Health System, Dover, USA; 2 Psychiatry, University of Connecticut Health, Farmington, USA

**Keywords:** 4-methylenedioxymethamphetamine (mdma), hallucinogen, lysergic acid diethylamide, major depression disorder, post stress traumatic syndrome, psilocybin, psychedelic-assisted psychotherapy, psychedelics

## Abstract

Psychedelic-assisted therapies have re-emerged as a subject of increasing scientific and clinical interest in psychiatry, particularly in the context of persistent treatment gaps for conditions such as treatment-resistant depression, post-traumatic stress disorder, and substance use disorders. Compounds, including psilocybin and 3,4-methylenedioxymethamphetamine, are being evaluated in controlled clinical trials and have demonstrated promising therapeutic effects when administered within structured psychotherapeutic frameworks. Emerging neurobiological evidence suggests that these agents may promote neural plasticity and facilitate cognitive and emotional flexibility, potentially enabling durable clinical improvement. Despite these advances, significant challenges remain, including regulatory uncertainty, methodological limitations in existing trials, ethical considerations related to patient vulnerability, and concerns regarding equitable access. This editorial examines the current state of psychedelic-assisted therapies, highlighting both their therapeutic potential and the critical considerations required for their responsible integration into mental health settings.

## Editorial

Psychiatry continues to experience significant therapeutic challenges, especially in patients with treatment-resistant depression, post-traumatic stress disorder (PTSD), and substance use disorders. Despite advances in psychopharmacology, many individuals fail to achieve sustained remission, highlighting the need for innovative treatment paradigms. In recent years, psychedelic-assisted therapies (PATs) have re-emerged as a topic of scientific and clinical interest. At one point in the past, it was sidelined due to sociopolitical factors rather than lack of scientific possibilities; compounds such as psilocybin, lysergic acid diethylamide (LSD), and 3,4-methylenedioxymethamphetamine (MDMA) are now being evaluated in controlled clinical trials [1]. This renewed interest reflects both growing dissatisfaction with conventional treatment models and advances in neuroscience that support alternative mechanisms of therapeutic change. As PATs approach potential clinical integration, it is imperative to examine their evidence base, regulatory status, ethical considerations, and practicality within existing mental health care systems.

Clinical trials and meta-analyses suggest that PATs may produce meaningful and sustained improvements in some of the selected mental health conditions. PAT is thought to act by transiently activating serotonergic and related neurobiological pathways that disrupt rigid brain networks, enhance neuroplasticity, and facilitate emotionally salient experiences, which, when integrated with psychotherapy, promote enduring psychological change [1]. Psilocybin-assisted therapy has shown meaningful reductions in depressive symptoms, including in patients with treatment-resistant depression, while MDMA-assisted therapy has shown promising outcomes for PTSD, and its effects persisted months after treatment completion [1,2]. These are encouraging findings given that treatment protocols typically involve a limited number of dosing sessions rather than prolonged medication use. Studies propose that psychedelics may facilitate therapeutic change by improving neuroplasticity and temporarily disrupting maladaptive patterns of neural connectivity associated with rigid cognitive and emotional processing [3]. This neurobiological flexibility, when combined with psychotherapy, may allow patients to engage with traumatic memories in novel and adaptive ways.

Despite promising findings, regulatory approval for PATs remains cautious. While the regulatory body has granted breakthrough therapy designation to psilocybin for major depressive disorder and MDMA for PTSD, recent regulatory deliberations have emphasized concerns related to trial methodology, expectancy effects, therapist variability, and long-term safety. These issues highlight the importance of conducting larger, carefully designed studies that follow consistent therapeutic approaches. Globally, the legal status of psychedelic compounds is still very patchy. Most of these substances remain classified as Schedule I, which makes research difficult and restricts access to them outside approved clinical trials. Schedule I refers to substances considered to have a high potential for abuse, no currently accepted medical use, and insufficient evidence of safety under medical supervision, which led to strict legal controls that curtailed research despite emerging scientific interest rather than clear evidence of therapeutic ineffectiveness. Without clear regulations and professional guidelines, introducing these treatments too early or without oversight could pose risks to patient safety and undermine public trust.

PATs differ from traditional medications because their effectiveness is closely tied to the therapeutic context in which they are delivered. Factors such as the patient’s mindset and the treatment setting play a crucial role in outcomes, emphasizing the importance of careful preparation, guided dosing sessions, and post-treatment integration therapy [4]. This approach challenges conventional psychiatric practice, which often focuses on brief medication management rather than deep psychotherapeutic engagement. The need for specialized therapist training, longer treatment sessions, and collaboration across disciplines also raises practical considerations about workforce availability, reimbursement, and the feasibility of scaling these therapies in real-world healthcare settings.

The growing use of PATs brings with it important ethical considerations. Because these treatments can heighten suggestibility, it is essential to maintain rigorous informed consent procedures and strict professional boundaries to protect patients from psychological harm [5]. PATs can increase suggestibility by loosening rigid thought patterns and self-focus, enhancing emotional openness and attention, and making individuals more receptive to therapeutic guidance and contextual cues during the session [5]. PATs are being investigated primarily for treatment-resistant depression, PTSD, anxiety related to serious illness, and substance use disorders; they are contraindicated in individuals with a personal or strong family history of psychotic or bipolar disorders and in uncontrolled cardiovascular disease, require precautions such as careful screening, medical monitoring, and structured psychotherapy, and are generally studied in adults aged ≥18 years, with use in children and adolescents typically avoided or considered contraindicated outside rigorously approved research settings. The rapid commercialization of psychedelic treatments also raises concerns that market forces could advance faster than the supporting evidence. Ensuring equitable access is another challenge: the costly nature of these therapies may limit availability to a privileged few, potentially widening existing disparities in mental healthcare. Finally, engaging ethically with indigenous and traditional knowledge systems, many of which have a long history of ceremonial psychedelic use, requires careful attention to avoid cultural appropriation and to foster respectful collaboration [5].

PATs offer a promising and potentially transformative approach in psychiatric care. Early research indicates they may benefit certain treatment-resistant conditions, supported by emerging insights from both neurobiology and psychotherapy. Yet, these therapies are not a cure. Their safe and effective integration into clinical practice will require rigorous research, clear regulatory frameworks, strong ethical safeguards, and careful attention to equitable access.

As psychiatry navigates this evolving landscape, a thoughtful, evidence-driven approach is essential. When implemented responsibly, PATs could become valuable adjuncts within a broader mental healthcare model, enhancing treatment options while upholding scientific rigor and clinical responsibility.
